# Bovine Respiratory Disease (BRD) in Post-Weaning Calves with Different Prevention Strategies and the Impact on Performance and Health Status

**DOI:** 10.3390/ani14192807

**Published:** 2024-09-28

**Authors:** Marina Madureira Ferreira, Bruna Santos, Agata Skarbek, Carley Mills, Hannah Thom, David Prentice, Craig McConnel, Francisco A. Leal Yepes

**Affiliations:** 1 Department of Animal Science, College of Agricultural and Life Sciences, Cornell University, Ithaca, NY 14853, USA; mm3373@cornell.edu; 2 Department of Veterinary Clinical Sciences, College of Veterinary Medicine, Washington State University, Pullman, WA 99164, USA; brussantos91@gmail.com (B.S.); agata.skarbek@wsu.edu (A.S.); carley.mills@wsu.edu (C.M.); hannah.thom@wsu.edu (H.T.);; 3 Elanco Animal Health Inc., Greenfield, IN 46140, USA; david.prentice@elancoah.com; 4 Department of Population Medicine and Diagnostic Sciences, College of Veterinary Medicine, Cornell University, Ithaca, NY 14853, USA

**Keywords:** BRD, *Mannheimia haemolytica*, metaphylaxis, weaning, health status

## Abstract

**Simple Summary:**

Bovine respiratory disease (BRD) is one of the major threats to the health and well-being of young calves. The disease affects long-term animal performance and has economic implications for cattle producers. Preventative strategies such as metaphylaxis have been shown to reduce the severity of BRD and improve performance and overall health status. However, the use of antibiotics in this fashion continues to come under scrutiny due to its possible link to antimicrobial resistance. The objective of our study was to compare a metaphylaxis to a vaccination strategy in high-risk dairy and dairy beef cross-bred calves. The use of metaphylaxis for BRD was superior to the vaccination strategy in this study.

**Abstract:**

Our study aimed to compare Bovine Respiratory Disease (BRD) morbidity, mortality, and growth in dairy and dairy beef cross-bred calves during the commingle period, 81–120 days of age, using two different BRD prevention strategies. The calves (n = 1799) were randomly assigned into groups: (1) Control (CON; received no vaccine or metaphylaxis); (2) Tulathromycin metaphylaxis (TUL; Increxxa^TM^, Elanco Animal Health Inc., Greenfield, IN, USA); and (3) *Mannheimia haemolytica* vaccine (VACC; Nuplura^®^ PH, Elanco Animal Health Inc., Greenfield, IN, USA). Calves were individually weighed three times during the study to estimate average daily gain (ADG). Deep nasopharyngeal swabs, thoracic ultrasonography, health scores, and treatment records were collected during the study. Ultrasound and health score results were not different across treatments. In this study, the TUL group had a lower cumulative BRD incidence than CON. The cumulative incidence and 95% CI of BRD during the commingle period, 81–120 days of age for CON, TUL, and VACC were 0.43 (0.38 to 0.47), 0.36 (0.38 to 0.40), and 0.39 (0.35 to 0.43), respectively. The ADG for CON, TUL, and VACC were 0.25 ± 0.15, 0.32 ± 0.15, and 0.17 ± 0.15 kg, respectively. There was no difference among the treatment groups for ADG. Management and environmental conditions were variable at this operation throughout the study period and likely impacted the calves. Earlier interventions may be needed when the BRD incidence is elevated in high-risk calves.

## 1. Introduction

Bovine respiratory disease (BRD) is one of the most common causes of morbidity and mortality in young dairy calves, especially during stressful events such as weaning and transition from individual to group housing [1]. Furthermore, BRD has negative economic consequences, evident not only in calf death loss or immediate treatment expenses, estimated at $42/case, but also in the long-term decline in performance following the disease event [2]. Dairy replacements affected with BRD are more likely to be removed from the herd prior to their first calving. Heifer calves with BRD have a reduced average daily gain (ADG), feed efficiency, and lower milk production during their first lactation [3,4,5]. Moreover, when performance impacts are considered, a single case of BRD in a young dairy heifer can cost over $250 [6]. Similarly, beef cattle are also affected by BRD, costing the industry between 800 and 900 million dollars annually and reducing animal performance and carcass quality [7,8]. Yet, addressing BRD continues to create significant challenges in calf management [9].

Bovine respiratory disease is a complex, multi-causal disease that includes several viral and bacterial pathogens along with numerous risk factors such as colostrum intake, housing, nutrition, environmental conditions, immune status, and age, all of which play a significant role in a calf’s susceptibility to the disease [3,9,10]. *Mannheimia haemolytica*, a Gram-negative bacterium, is one of the predominant organisms involved in BRD [11,12,13]. Although it is a natural commensal in the upper respiratory tract, it becomes an opportunistic pathogen responsible for BRD clinical signs in affected animals under challenging conditions.

In the United States, approximately one-fifth of beef production comes from animals of dairy herd origin, and it keeps growing [14]. The widespread use of sexed semen in the dairy industry has allowed producers to be more selective in choosing genetically superior animals to obtain their supply of dairy replacement heifers. Sexed semen opened new breeding options for the remaining cows, using beef semen to increase beef production with dairy cross-bred calves [15,16]. Beef on dairy cross-bred animals are generally more desired than Holsteins because they bring more value to producers and processors. Therefore, beef-on-dairy calves are a growing trend in the cattle industry [17,18]. Most beef-on-dairy calves are transported from their dairy of origin to calf-raising facilities at an early age, often within three days of birth. These calves are frequently at a high risk for respiratory infections due to inadequate pre-shipment management, increased stress, and exposure to several new pathogens during transportation or upon arrival at a new facility [19,20].

To help control or minimize BRD, immunization and metaphylaxis are regularly implemented upon the arrival of animals at the calf-raising facilities [21]. Metaphylactic antimicrobial medications have been employed in about half of all calf-raising facilities to prevent BRD when the host is more susceptible [22,23]. Tulathromycin is a macrolide antibiotic frequently used for metaphylaxis treatment of BRD. It has shown superior performance compared to many other BRD drugs used for the same purpose by reducing BRD morbidity and mortality, and it may increase ADG [24,25].

Therefore, the primary objective of this study was to compare BRD morbidity and mortality in dairy and dairy beef cross-bred calves at a facility that was reporting a high incidence (>25%) of BRD during the commingle using two different BRD prevention strategies: (1) Tulathromycin metaphylaxis, at 100 mg/mL concentration (TUL; Increxxa^TM^, Elanco Animal Health Inc., Greenfield, IN, USA), and (2) Vaccine with a single dose of *Mannheimia haemolytica* bacterial extract toxoid (VACC; Nuplura PH^®^, Elanco Animal Health Inc., Greenfield, IN, USA). The secondary outcomes explored in our study were the effects of these two interventions on growth, calf health scores, and thoracic ultrasound. We hypothesized that both prevention strategies would reduce BRD morbidity and mortality and result in better health and performance than control calves.

## 2. Materials and Methods

### 2.1. Sample Size Calculations

The sample size was calculated based on the effects of metaphylaxis in reducing BRD cases by approximately 50% in the post-treatment period [26]. Based on a 25% baseline BRD morbidity and a 50% reduction in BRD cases, power calculations indicate that a group size of 152 per treatment would be sufficient to detect a difference between metaphylaxis and control calves. However, the reduction in BRD secondary to *M. haemolytica* vaccination is not well-defined. We hypothesized that vaccination would reduce morbidity by 25%. Given a baseline of 25% BRD morbidity post-commingling, a 25% reduction would result in about 18% morbidity. The number of animals required to show a difference between 25% and 18% morbidity given a two-sided test, alpha at 0.05, and power of 0.80 is approximately 540 animals (JMP 17.2, JMP Statistical Discovery LLC, Cary, NC, USA). We targeted 600 calves per treatment group, accounting for some loss of follow-up during the study period.

### 2.2. Animals, Housing, and Management

All procedures were approved by the Washington State University Institutional Animal Care and Use Committee (ASAF# 7090). The study was conducted on a commercial calf ranch in Central Washington from July to December 2022. The ranch received dairy and dairy beef cross-bred calves from different dairies several times each week. Calves arrived at the facility within one to three days of age. Upon arrival, all the animals enrolled in the study were identified using a color ear tag with sequential ID numbers and Radio Frequency Identification (RFID).

Calves were placed in individual wood hutches with straw bedding (2.4 m × 1.2 m × 1.2 m) from arrival until approximately 80 days of age. The calves followed a step-up/step-down feeding regimen. They received 3.8 L/d of milk replacer from day 1 to 14, then it increased to 5.6 L/d from day 15 to 50, returning to 3.8 L/d from day 51 to 55, followed by feeding 0.9 L/d milk replacer from d 56 to 60. Calves were weaned at 60 days of age and remained in their hutches until around 80 days of age. Grain and water were offered ad libitum upon arrival and throughout the study. All calves transitioned to a grower diet per standard operating procedures on this operation.

Upon removal from the hutches, calves were individually weighed and moved into the commingle pens in groups of approximately 35 animals (18.2 m × 9.1 m). The group pens were filled over time, and calves were housed in these pens by the treatment group for six weeks (81–120 days of age), which was the observation period for this study. In total, calves were housed by treatment across 54 pens. In the group pens, animals were fed total mixed, silage-based diet ad libitum.

**Figure 1:**
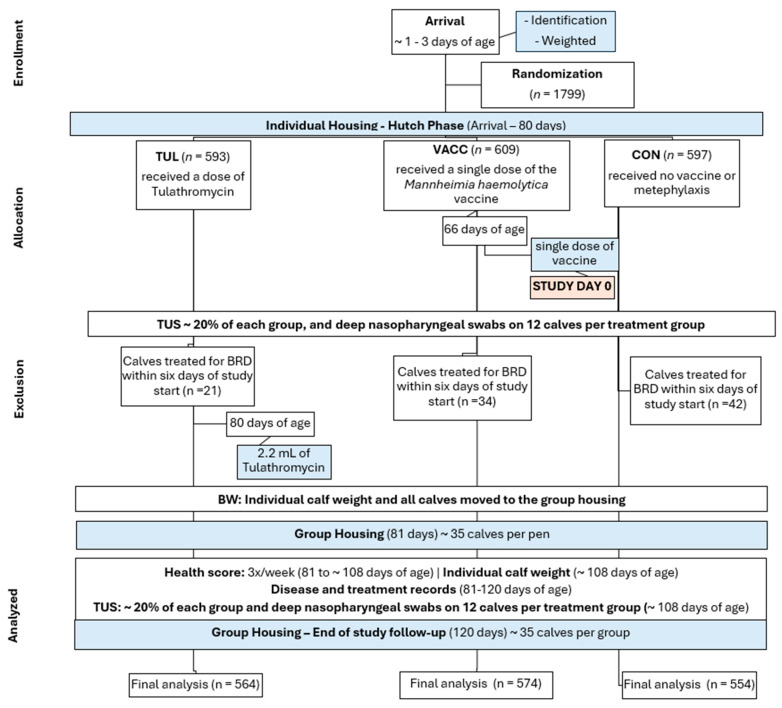
A flowchart describing the study design and the time for each event during the trial.

Our study population consisted of 1799 dairy and dairy beef cross-bred calves randomly assigned upon arrival to the facility into one of the three treatment groups, controlled for sex and breed using PROC SURVEYSELECT (SAS 9.4, SAS Institute Inc., Cary, NC, USA). Treatment groups were: (1) Control (CON; received no vaccine or metaphylaxis; n = 597); (2) Tulathromycin metaphylaxis (TUL; Increxxa^TM^, Elanco Animal Health Inc., Greenfield, IN, USA; n = 593), a 2.2 mL dose given on the day of commingle, approximately 80 days of age; and (3) *M. haemolytica* vaccine (VACC; *Mannheimia haemolytica* bacterial extract-toxoid Nuplura PH^®^, Elanco Animal Health Inc., Greenfield, IN, USA; n = 609)—a single dose, 2 mL, subcutaneously was given around 14 days prior to movement from hutches, approximately 66 days of age, which is the start of the study. The tulathromycin dose was based on average body weights at weaning at the facility the month before the start of the study. Figure 1 shows a diagram of the study flow.

### 2.3. Weights and Average Daily Gain

Calves’ body weight (BW) was measured using a portable scale (Optima Scale Livestock Scale—Capacity, 0.5-lb. Increment Display, Model# OP-930, Rancho Cucamonga, CA, USA) within three days of arrival by research staff and recorded in Excel (v 16.0, Microsoft Corp, Redmond, WA, USA). Calves were individually weighed when moved to the group pens around 80 days of age and then again at the end of the first four weeks in the group housing (~108 days of age). The ADG was calculated as follows:

$$\frac{BW\;at\;108\;days\;of\;age-BW\;at\;80\;days\;of\;age}{days\;between\;the\;two\;BW}$$

### 2.4. Thoracic Ultrasound and Nasal Swab

A subset of calves (~20%) in each group underwent thoracic ultrasound (TUS) examination on day 0 of the study, simultaneously with vaccination (66 days of age). The ultrasound was conducted by trained research staff who were blinded to the treatment groups. They were present on the farm only during the evaluation days and did not have access to the treatment groups. Any calf with a TUS score ≥ 3 at the initial TUS was considered consolidated and thus excluded from a second ultrasound, since lung consolidation can persist despite treatments and the resolution of clinical signs [27,28]. Calves with a TUS score < 3 at the first evaluation underwent a second TUS approximately four weeks after moving into the group housing, around 108 days of age. The TUS exams were performed using an IBEX Pro portable ultrasound (E.I. Medical Imaging, Loveland, CO, USA) with an 8.5 MHz linear array transducer. The ultrasound evaluation was scored using a standardized and validated five-point scale [29].

Concurrently, deep nasopharyngeal swabs were carried out on 12 calves per treatment group from different pens to have a representative sample from the study population. Briefly, the calf was restrained, and two sterile swabs (Puritan PurFlock Ultra^®^ Flocked Swabs, Puritan Medical Products, Guilford, ME, USA) were inserted 3–6 cm into the calf’s nostril being careful to avoid touching the tip of the swabs to the exterior of the calf’s nares. The swabs were rotated against the wall of the nostril for five turns and then removed from the nostril. The same swabs were inserted into the other nostril, where the procedure was repeated. The two swabs were placed in a tube pre-labeled with calf ID and date, placed in ice, and transported to a freezer at −80 °C until further analysis.

The DNA from the swabs was extracted using ZymoBIOMICS 96 DNA Kit (#D4309, Zymo Research Corporation, Irvine, CA, USA) following manufacturer instructions. The qPCR assay targeting respiratory bacterial *M. haemolytica* was based on previous publications [30]. All the samples were run in duplicates, and all the plates included a housekeeping gene (GAPDH) and a positive *M. haemolytica* sample obtained from the Washington Animal Disease Diagnostic Lab (WADDL, Pullman, WA, USA). The DNA extraction and processing of the swabs were performed by a research staff blinded to the treatment groups.

### 2.5. Health Scores and Treatment Records

Study personnel examined and scored the calves three times weekly during the first four weeks in the group housing (81–108 days of age), using a scoring system adapted from The Wisconsin Calf Health Scoring System (Supplementary Materials). The maximum score any calf could receive at a single observation was 9 (Nose on a scale of 0–3; Ear on a scale of 0–3; and Eyes on a scale of 0–3). A calf with a score ≥ 5 on the Wisconsin Calf Scoring Chart is commonly treated with antibiotics for BRD.

Diagnosis and treatment records were kept in the ranch data software (Bovisync LLC, Fon du Lac, WI, USA) as they were prior to the start of the study. In this study, ranch personnel were blinded to the treatment groups, health scores, TUS, and deep nasopharyngeal swabs. Ranch standard operating procedures and treatment protocols were not adjusted during this trial, with the exception that metaphylaxis of any kind (injection, feed, water) was not allowed throughout the trial. Animal observation and live animal phase were completed six weeks after the commingle at 120 days of age.

### 2.6. Weather Data

The weather (e.g., maximum and minimum daily air temperature, humidity, wind speed, and solar radiation) data during the study period were collected using a Washington State Weather station located 2.6 miles away from the calf ranch. The time-series graphs were created using Proc SGPLOT (SAS 9.4).

### 2.7. Statistical Analysis

There were 1799 calves available for inclusion in the analysis for the study at the time of randomization. Table 1 shows descriptive statistics of calves eligible for enrollment. The treatment of diseases by the farm personnel was not restricted at any time during the study. However, the study protocol specified that any calf treated with antibiotics six days prior to day 0 of the study, which was marked by the vaccination day, would be excluded from the analysis (regardless of group). The exclusion was defined based on the duration of the antimicrobial effect, the period that the drug is no longer available to contribute to treatment success. Post-treatment Interval (PTI) studies for BRD suggest 7 days may be a default PTI [31]. There were 42 CON, 21 TUL, and 34 VACC calves treated by ranch personnel within six days of the study start. Moreover, calves that died before the completion of the study were also excluded. Thus, 1692 calves were included in the final analysis (554, 564, and 574 CON, TUL, and VACC, respectively). In this study, the individual calf was considered the experimental unit. The Cochran–Mantel–Haenszel tests were performed using PROC FREQ of SAS (SAS 9.4) for differences in categorical variables such as health score observations, deep nasopharyngeal swabs, and thoracic ultrasonography. The BW and ADG of animals that died during the study period were excluded from the analysis. One-way ANOVA was used to determine the differences in continuous variables such as the number of BRD treatments, average weight at arrival at the calf ranch, and ADG. The differences in BW at 80 and 108 days of age among treatments were analyzed with repeated measures ANOVA using PROC Mixed in SAS 9.4. Five covariance structures were tested for the differences in BW outcome (simple, compound symmetry, autoregressive order 1, Toeplitz, and unstructured), and the covariance structure with the lowest Akaike’s information criterion was selected. The Pen and Arrival Group were included as Random effects. Treatment and BW at arrival were included as a fixed effect, and the REPEATED statement was used for the time variable. Other plausible fixed effects terms (e.g., sex, breed, farm of origin, and weight at arrival) were tested and not included in the final model if the *p*-value ≥ 0.05. The treatment × time interaction was forced in the model. Other plausible interaction terms were tested and not included in the final model if the *p*-value ≥ 0.05. Tukey’s post hoc test was used for multiple comparison correction of *p*-values for all pairwise comparisons of least-squares means. Normality and homoscedasticity of residuals were visually evaluated for the model fit. Significance for all analyses was declared if *p* < 0.05.

Logistic regression models using PROC GLIMMIX and PROC LOGISTIC were used to investigate potential associations between the treatment groups and health scores, thoracic ultrasonography, deep nasopharyngeal swabs, and BRD mortality. Other plausible fixed effects terms (e.g., sex, breed, farm of origin, and weight at arrival) were tested and not included in the final model if the *p*-value ≥ 0.05. The Pen and Arrival Group were incorporated as a random effect in the models. Then, odds ratios were calculated for these associations.

Survival analysis was performed to determine the age at first BRD treatment by ranch personnel in the group housing. The cumulative incidence of BRD among the treatment groups was estimated using PROC LIFETEST and Kaplan–Meier methods.

## 3. Results

### 3.1. Respiratory Morbidity, Mortality, and Growth

The overall probability of a calf developing BRD within the first 80 days at this facility was 98.2%. Prior to the start of the study period, mortality within the first 80 days was 14.3%, and it was not different across the treatment groups (Table 2).

**Figure 2:**
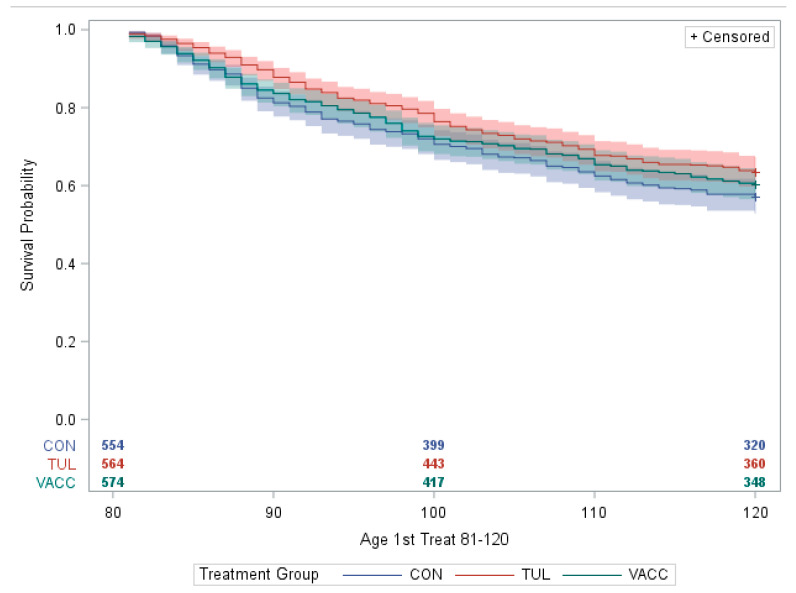
Survival analysis of BRD during group housing at 81–120 days of age. The shaded areas represent the 95% CIs. + Censored data are relative to calves who may not experience BRD until the end of the study observation period, 81 to 120 days of age. Treatments: (1) CON (no vaccination against *M. haemolytica* or metaphylaxis); (2) TUL (2.2 mL at commingle, at 80 days of age); and (3) VACC (*M. haemolytica* vaccine at approximately 14 days prior to commingle, at 66 days of age).

The survival analysis is shown in Figure 2 and illustrates the probability of calves developing BRD, determined by the first BRD treatment by ranch personnel during the group housing, 81–120 days of age. The survival data present censored observations relative to those calves who may not experience BRD before the end of the study. The cumulative incidence and 95% CI of BRD during 81–120 days for CON, TUL, and VACC groups were 0.43 (0.38 to 0.47), 0.36 (0.38 to 0.40), and 0.39 (0.35 to 0.43), respectively. The TUL group had a lower probability (*p* = 0.02) of developing BRD post-commingle (81–120 days of age) than the CON group. In contrast, there was no difference between the CON and VACC groups (*p* = 0.55) or TUL and VACC (*p* = 0.28) groups (Figure 2). Moreover, as shown in Table 2, there were no mortality differences among the groups during the study period (*p* = 0.40). However, we detected a significant effect of the arrival group on the incidence of respiratory diseases (*p* = 0.002) that had no pattern related to the arrival date at the operation.

Moreover, the CON had 1.2 (95% CI: 0.9 to 1.5) and 1.3 (95% CI: 1.0 to 1.5) greater odds of being treated for pneumonia than VACC and TUL calves (*p* = 0.08). Similarly, the CON had 1.2 (95% CI: 0.6 to 2.2) and 1.4 (95% CI: 1.0 to 1.5) greater odds of mortality than VACC and TUL calves during the study period (*p* = 0.59).

**Figure 3:**
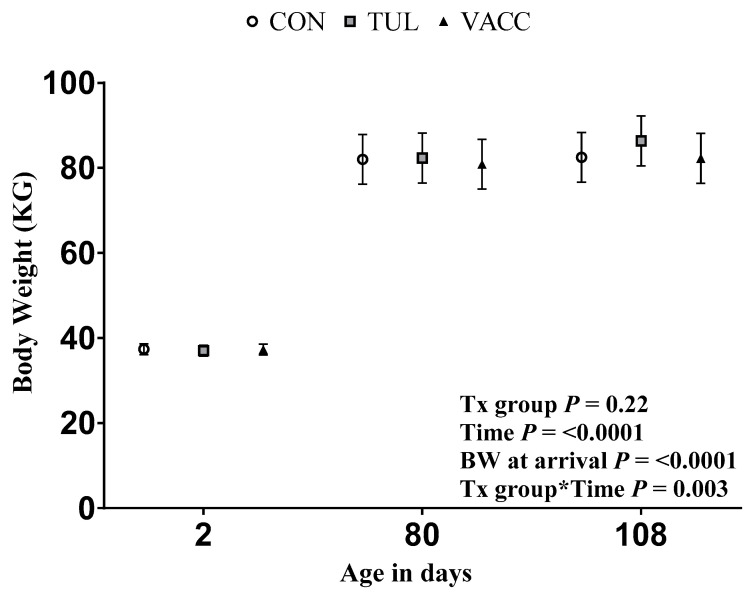
Least-squares means of repeated measures ANOVA for BW in KG (95% CI) in four weeks in the group housing (81–108 days of age). Treatments: (1) CON (no vaccination against M. haemolytica or metaphylaxis); (2) TUL (2.2 mL at commingle, at 80 days of age); and (3) VACC (M. haemolytica vaccine at approximately 14 days prior to commingle, at 66 days of age). The model included the treatment group, time, and BW at enrollment, with the arrival group and pen included as a random effect. The different superscript letters indicate a *p*-value ≥ 0.05 on Tukey’s post hoc test from multiple comparison correction of least-squares means.

There was no difference in BW at arrival among the treatment groups (*p* = 0.60, Table 1). The least-squares means of BW (Kg ± SE) are shown in Figure 3. At 80 days of age, CON, TUL and VACC calves BW were 81.9 ± 2.9, 82.3 ± 2.9, and 80.8 ± 2.9 kg, respectively (*p* = 0.54). Four weeks later, in the grouping house (108 days), there was no difference in BW between treatment groups (*p* = 0.36). TUL (86.3 ± 2.9), CON (82.4 ± 2.9), and VACC (82.2 ± 2.9) The ADG (LSM ± SE in Kg) for CON, TUL, and VACC groups were 0.25 ± 0.15, 0.32 ± 0.15, and 0.17 ± 0.15, respectively. There was no difference among the treatment groups for ADG (*p* = 0.37). On average, calves among all treatment groups gain 1.97 (1.1 to 2.9) Kg of BW between 80 and 120 days of age.

### 3.2. Thoracic Ultrasound and Nasopharyngeal Swabs

The thoracic ultrasonography results are shown in Table 3. At the first ultrasound, which occurred on the day of vaccination, 66 days of age, 35% of the calves had ultrasound scores ≥ 3, indicating one or more lobes were consolidated, and these were not different across treatment groups (*p* = 0.77). Calves with consolidated lungs (score ≥ 3) on the first ultrasound were not subjected to a second thoracic ultrasound. On the second ultrasound, four weeks after movement into the group housing, 37.5% of calves were consolidated, with no difference observed between treatment groups (*p* = 0.86).

The results of nasopharyngeal swabs are shown in Table 4. *M. haemolytica* was detected in 54% of calves sampled among the treatment groups and CON groups on the day of vaccination (*p* = 0.48) and in 97% of the calves after four weeks in the group housing, with no difference observed between treatment groups (*p* = 0.99).

### 3.3. Calf Scoring Results

The health score assessment results are summarized in Table 5. There were no differences in the health scores among the treatment groups (*p* = 0.50). However, the health scores changed over the study time (*p* < 0.0001) and were different among the pens (*p* < 0.0001) with no particular pattern.

### 3.4. Weather Conditions

**Figure 4:**
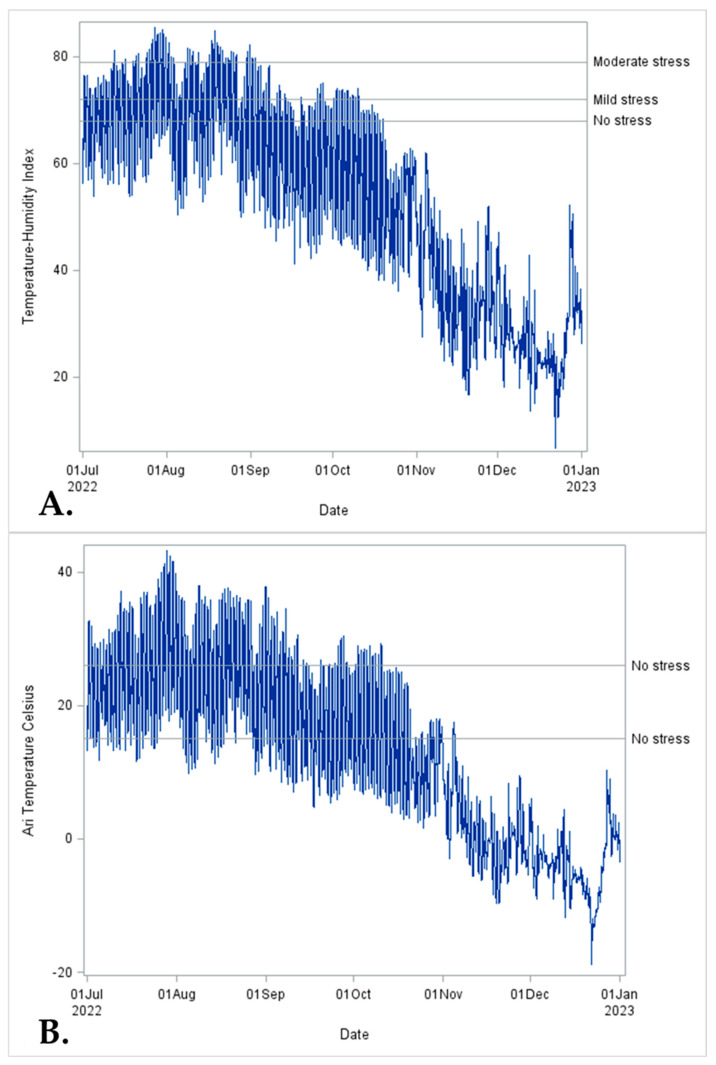
The series plot is from July to December 2022, Washington State University Ag-Weather station located in Mabton, WA, for temperature–humidity index (THI) and air temperature (°C). (**A**): The solid lines represent different thresholds for heat stress: no stress (THI < 68), mild stress (THI 72–78), moderate stress (THI 79–89), and severe stress (THI ≥ 90). (**B**): The solid lines represent the thermoneutral zone for young calves; below the thermoneutral zone, calves are under cold stress: no stress 15 °C to 26 °C.

The temperature–humidity index (THI) was used to determine the levels of heat stress, as shown in Figure 4A. These levels include no stress (THI < 68), mild stress (THI 72–78), moderate stress (THI 79–89), and severe stress (THI ≥ 90) [32]. Figure 4B illustrates the thermoneutral zonefor. There were extreme temperature changes during the study period. In the early stages of the study, calves experienced heat stress upon arrival at the calf ranch. In contrast, during the final phase, they experienced cold stress around weaning. The range of temperatures for the comfort zone becomes 15 °C to 26 °C [33,34].

## 4. Discussion

Our study aimed to evaluate the incidence of BRD in dairy and dairy–beef cross-bred calves during the commingle groups with two BRD prevention strategies. In addition, we analyzed the effects of these interventions on growth, calf health scores, and thoracic ultrasound.

The use of antimicrobials as a metaphylactic treatment in food production animals is controversial since it may increase antimicrobial resistance [35]. Therefore, using vaccines as an alternative to reduce BRD incidence in high-risk calves is desirable. Vaccination is a well-documented effective strategy to reduce BRD in cattle [36]. The administration of a fraction of the antigen, usually the membrane proteins, initiates an innate immune response, followed by the adaptative immune response, that will establish specific immunological memory. When the animal is exposed to the same pathogen in the future, specific memory cells are activated and serve as a fast front-line defense. Therefore, it is crucial to implement vaccines strategically before the host is exposed to the pathogen. However, in our study, we collected a deep nasopharyngeal swab from the calves at the time of vaccination, approximately 66 days of age, and we detected *M. haemolytica* from the calves before they were inoculated with the vaccine. Furthermore, other stressful factors, such as nutritional challenges, feed changes, adverse weather conditions, and commingling, can contribute to immune suppression and the calves’ ability to respond to the vaccine [37].

For a vaccine to be considered effective, it must be able to reduce BRD morbidity and mortality and improve weight gain [38]. However, the efficiency of vaccines in high-risk calves is compromised due to stress factors that induce immune dysfunction. A previous study reported that beef calves experience an acute-phase protein response within a two-week period after vaccination, which can result in reduced ADG. In the study, heifers that received an *M. haemolytica* vaccine had a lower ADG than the control [39].

Similarly, metaphylaxis is routinely used in high-risk calves, particularly following a stressful event like commingling, when animals become more susceptible to new infections. Tulathromycin is a long-acting antibiotic that maintains elevated drug concentrations in the lungs for several days following a single subcutaneous injection, and it is commonly used for metaphylaxis [40]. From the operational perspective of large cattle operations, a single injection with a long-acting antibiotic optimizes the prevention and treatment of BRD. In previous studies, tulathromycin was reported as a superior drug when compared to enrofloxacin or oxytetracycline for the management of BRD in high-risk stocker cattle [28,41]. In the current study, TUL calves were less likely to develop BRD between 81 and 120 days than CON calves.

Moreover, metaphylaxis with tulathromycin has been shown to improve the performance of high-risk feedlot cattle compared to a pentavalent modified-live virus respiratory vaccine (MLV). Calves that received antimicrobial metaphylaxis were 13.4 kg heavier on day 56 post-treatment than those that did not receive metaphylaxis [42]. In our finding, there was a numeric difference but no statistical difference in the ADG of the TUL group when compared with the CON or VACC group. Moreover, we observed that calves gain a small amount of weight on average between 81–120 d of age, regardless of the treatment group. All calves enrolled in the study were exposed to severe weather conditions concomitant with other stressors such as inadequate colostrum intake, transportation at an early age to the calf ranch, weaning, and commingling [43], which may have a direct impact on the ADG.

Severe weather conditions increase BRD morbidity and mortality and reduce ADG [43,44,45,46]. Cold stress affects the mucociliary clearance and macrophage response, reducing the respiratory protective barrier and predisposing cold-exposed calves to respiratory tract infection [47]. The ADG in the four weeks of commingling for all treated and control calves in our study were exceptionally low among all groups, 0.25 ± 0.15, 0.32 ± 0.15, and 0.17 ± 0.15, respectively, for the CON, TUL, and VACC groups. Our study observed poor performance among all the groups compared to the expected ADG of around 0.9 kg/d for calves during the first month post-weaning [48]. It is plausible that the weather during the study period contributed to these results. Calves experiencing cold stress increase their feed intake and metabolic rate, using the energy to thermoregulate and maintain their core body temperature. However, the cold stress negatively impacts the calves’ digestive function, resulting in lower nutrient digestibility [49]. The maintenance energy requirement increases by 2.01 kcal/kg ^0.75^ per day for each degree above or below the thermoneutral zone (15 °C to 26 °C) to thermoregulate [50]. Therefore, despite the increase in feed intake, it is not equivalent to heat production, resulting in lower growth [49,51] and making calves more susceptible to diseases [52].

Furthermore, in dairy calves, lung lesions are associated with a lower body weight and reduced ADG preweaning [53], and it is plausible that this is similar in dairy beef cross calves. Previous studies reported that calves with more than one lobe consolidated using the consolidation cut-off of ≥ 3 cm lesion had a decreased ADG [54]. Moreover, Rhodes et al., (2021) found that calves with TUS scores ≥ 3 had a predicted ADG of 126 g/d lower than unaffected calves (TUS score ≤ 1, no consolidation) across the preweaning period, and the weight predictions were even lower when the consolidation was detected later, close to the weaning period [55]. These calves detected later have less time to recover before weaning, a stressful period for the calf, where the immune system is affected by diet changes and other stressors, increasing the susceptibility for BRD. In our study, lung consolidation was observed among treatments and CON before and after commingling in many calves. This suggests that BRD was still present in the calves despite the interventions (e.g., TUL and VACC) and may explain the poor ADG during the study.

The deep nasopharyngeal swabs were performed to detect *M. haemolytica*, the most common pathogen associated with BRD. However, due to its multi-etiological complexity, one potential reason for the persistence of the infection after interventions could be co-infection with other pathogens, such as *Pasteurella multocida, Histophilus somni,* bovine viral diarrhea virus (BVDV), bovine respiratory syncytial virus (BRSV), and bovine herpes virus (BHV). Co-infections can complicate treatment and contribute to persistent infections.

Furthermore, despite the protective effect of the TUL group from BRD, calves were housed in group pens adjacent to the CON group, where calf-to-calf contact was inevitable through the fence. The CON group could potentially serve as a reservoir, acting as a source of reinfection for the calves even after initial treatment. In addition, calves among all groups were constantly challenged by harsh environmental conditions, increasing their susceptibility to common BRD pathogens. Another plausible explanation is the potential for antimicrobial resistance. It is well-known that bacteria have the capacity to develop multiple mechanisms to evade the host’s immune system and resist treatment effects [56].

Recently, the published literature has found multidrug resistance strains of *M. haemolytica* in high-risk cattle using tulathromycin as a metaphylaxis strategy and with other long-acting antimicrobial drugs, such as tildipirosin. The detection of *M. haemolytica* from deep nasopharyngeal swabs in high-risk animals increased from arrival to the revaccination period, and they also reported that multi-drug-resistant strains of the bacteria can be highly prevalent in raising facilities [41,56,57]. These results were in accordance with our findings, where *M. haemolytica* was detected in 54% of calves on the day of vaccination and then in 97% of the calves four weeks after commingle. Besides that, using the same antimicrobial class for both metaphylaxis and treatment may contribute to the developing multidrug resistance in the operation.

Our research was conducted in a large commercial calf operation; however, it had some limitations. Upon arrival, the calves were already at a considerable risk of developing diseases due to numerous factors, such as coming from different origins, insufficient colostrum intake, long hours of transportation, dehydration, and stress. These factors play a significant role in the calves’ lower performance and immune response, ultimately resulting in a reduced response to treatments. In addition, we were not able to analyze the differences in the underlying causes of mortality among the treatment groups due to limitations in frequency and the oversight of postmortem evaluations conducted by farm personnel.

## 5. Conclusions

On this calf operation, the use of a *M. haemolytica* vaccine (Nuplura) pre-commingling did not reduce BRD morbidity or mortality in dairy and dairy–beef cross-bred calves. The metaphylactic treatment with tulathromycin did result in calves with a reduced cumulative incidence of BRD compared to those given vaccine or control calves. Deep nasopharyngeal swabs collected before the vaccination and metaphylactic treatment suggest that the overall population was already exposed to *Mannheimia haemolytica*. Therefore, vaccination with *M. haemolytica* may have been administered too late to provide protection.

## Figures and Tables

**Table 1:** Description of the study population eligible for enrollment by calf characteristics and treatment group.

		^1^ Treatment Groups			
Characteristics		CON	TUL	VACC	*p*-Value
Sex	Female	225	221	227	0.98
	Male	372	372	382	
Breed	Angus	224	227	231	0.90
	Charolais	169	162	168	
	Holstein	72	71	69	
	Limousine	132	133	141	
^2^ Body weight at arrival ± SE, (kg)		37.3 ± 0.5	37.0 ± 0.5	37.1 ± 0.5	0.60

^1^ Treatments: (1) CON (no vaccination against *M. haemolytica* or metaphylaxis); (2) TUL (2.2 mL at commingle, at 80 days of age); and (3) VACC (*M. haemolytica* vaccine at approximately 14 days prior to commingle, at 66 days of age). All the data are presented as total counts unless otherwise stated. ^2^ The BW at arrival is presented as the least-squares means of ANOVA in Kg ± SE.

**Table 2:** Respiratory morbidity (based on treatment counts) and mortality.

	Treatment Groups ^1^			*p*-Value
Event	CON	TUL	VACC	Tx
Calves included in the analysis	554	564	574	
LSM ± SE BRD treatment 81–120 days ^2^	0.53 ± 0.03	0.45 ± 0.03	0.47 ± 0.03	0.10
Mortality (n, %)	35 (6.3%)	26 (4.6%)	35 (6.1%)	0.40

^1^ Treatments: (1) CON (no vaccination against *M. haemolytica* or metaphylaxis); (2) TUL (2.2 mL at commingle, at 80 days of age); and (3) VACC (*M. haemolytica* vaccine at approximately 14 days prior to commingle, at 66 days of age). ^2^ Treatment group was included in the model, with the arrival group and pen included as a random effect.

**Table 3:** Ultrasound results for lung consolidation detection using a five-point scale previously validated of a subset of calves before and post-commingle using the Cochran–Mantel–Haenszel test for differences in categorical variables.

		^1^ Treatment Groups				
					*p*-Value	
Time of Ultrasound Collection		CON	TUL	VACC	Tx	Time
^2^ 66 days of age	Score < 3	71 (67.6%)	80 (64.5%)	70 (63.1%)		
	Score ≥ 3	34 (32.4%)	44 (35.5%)	41 (36.9%)		
					0.77	0.001
^3^ 108 days of age						
	Score < 3	30 (61.2%)	39 (65.0%)	29 (60.4%)		
	Score ≥ 3	19 (38.2%)	21 (35.0%)	19 (39.6%)		

^1^ Treatments: (1) CON (no vaccination against *M. haemolytica* or metaphylaxis); (2) TUL (2.2 mL at commingle, at 80 days of age); and (3) VACC (*M. haemolytica* vaccine at approximately 14 days prior to commingle, at 66 days of age). ^2^ Calves were vaccinated at approximately 66 days of age, two weeks before moving into the group housing. ^3^ Calves were evaluated after four weeks in the group housing, approximately 108 days of age.

**Table 4:** Deep nasopharyngeal swab results for detection of *M. haemolytica* of a subset of calves before and post-commingle using the Cochran–Mantel–Haenszel test for differences in categorical variables.

		^1^ Treatment Groups				
Time of Swab Collection					*p*-Value	
		CON (n, %)	TUL (n, %)	VACC (n, %)	Tx	Time
^2^ 66 days of age	Positive	8 (66.6%)	7 (53.8%)	5 (41.6%)		
	Negative	4 (33.3%)	6 (46.2%)	7 (58.3%)		
					0.48	0.97
^3^ 108 days of age						
	Positive	10 (100.0%)	11 (91.6%)	11 (100%)		
	Negative	0 (0.0%)	1 (8.4%)	0 (0.0%)		
	Died	2	1	1		

^1^ Treatments: (1) CON (no vaccination against *M. haemolytica* or metaphylaxis); (2) TUL (2.2 mL at commingle, at 80 days of age); and (3) VACC (*M. haemolytica* vaccine at approximately 14 days prior to commingle, at 66 days of age). All the data are presented as total counts otherwise stated. ^2^ Calves were vaccinated at approximately 66 days of age, two weeks before moving into the group housing. ^3^ Calves were evaluated after four weeks in the group housing, approximately 108 days of age.

**Table 5:** Calf health scores using the modified Wisconsin Scoring System three times a week for four weeks following commingle (81–108 days of age), using the Cochran–Mantel–Haenszel test for differences in categorical variables.

Total Counts of Eye, Nose, and Ear Scores	^1^ Treatment Groups				*p*-Value
	CON	TUL	VACC	Tx	Time
Score < 5	6915 (32.4%)	7040 (32.9%)	6983 (32.7%)	0.50	<0.0001
Score ≥ 5	141 (0.7%)	136 (0.7%)	169 (0.8%)		

^1^ Treatments: (1) CON (no vaccination against *M. haemolytica* or metaphylaxis); (2) TUL (2.2 mL at commingle, at 80 days of age); and (3) VACC (*M. haemolytica* vaccine at approximately 14 days prior to commingle, at 66 days of age).

## Data Availability

The data are available upon request.

## Supplementary Materials

The following supporting information can be downloaded at: https://www.mdpi.com/article/10.3390/ani14192807/s1, Figure S1. The scoring system used in the study was adapted from the Calf Health Scoring Chart from the University of Wisconsin, a total score of 9 was possible (nasal discharge, eyes, and ears).

## References

[1] USDA (2021). Dairy 2014, “Trends in Dairy Cattle Health and Management Practices in the United States, 1991–2014” USDA–APHIS–VS–CEAH-NAHMS. Fort Collins, CO #711.0821 2021.

[2] Dubrovsky S.A., Van Eenennaam A.L., Aly S.S., Karle B.M., Rossitto P.V., Overton M.W., Lehenbauer T.W., Fadel J.G. (2020). Preweaning Cost of Bovine Respiratory Disease (BRD) and Cost-Benefit of Implementation of Preventative Measures in Calves on California Dairies: The BRD 10K Study. J. Dairy Sci..

[3] Peel D.S. (2020). The Effect of Market Forces on Bovine Respiratory Disease. Vet. Clin. N. Am. Food Anim. Pract..

[4] Buczinski S., Achard D., Timsit E. (2021). Effects of Calfhood Respiratory Disease on Health and Performance of Dairy Cattle: A Systematic Review and Meta-Analysis. J. Dairy Sci..

[5] Stanton A.L., Kelton D.F., LeBlanc S.J., Wormuth J., Leslie K.E. (2012). The Effect of Respiratory Disease and a Preventative Antibiotic Treatment on Growth, Survival, Age at First Calving, and Milk Production of Dairy Heifers. J. Dairy Sci..

[6] Overton M.W. (2020). Economics of Respiratory Disease in Dairy Replacement Heifers. Anim. Health Res. Rev..

[7] (2020). Preview: Economic Effects of Bovine Respiratory Disease. Journal of Animal Science.

[8] Blakebrough-Hall C., McMeniman J.P., González L.A. (2020). An Evaluation of the Economic Effects of Bovine Respiratory Disease on Animal Performance, Carcass Traits, and Economic Outcomes in Feedlot Cattle Defined Using Four BRD Diagnosis Methods. J. Anim. Sci..

[9] Aly S.S., Love W.J., Blanchard P.C., Crossley B., Van Eenennaam A.L., Lehenbauer T.W. (2021). Etiology and Risk Factors for Bovine Respiratory Disease in Pre-Weaned Calves on California Dairies and Calf Ranches. Prev. Vet. Med..

[10] Cummings D.B., Meyer N.F., Step D.L. (2022). Bovine Respiratory Disease Considerations in Young Dairy Calves. Vet. Clin. N. Am. Food Anim. Pract..

[11] Snyder E., Credille B. (2020). Mannheimia Haemolytica and Pasteurella Multocida in Bovine Respiratory Disease. Vet. Clin. N. Am. Food Anim. Pract..

[12] Booker C.W., Abutarbush S.M., Morley P.S., Jim G.K., Pittman T.J., Schunicht O.C., Perrett T., Wildman B.K., Fenton R.K., Guichon P.T. (2008). Microbiological and Histopathological Findings in Cases of Fatal Bovine Respiratory Disease of Feedlot Cattle in Western Canada. Can. Vet. J..

[13] Poonsuk K., Kordik C., Hille M., Cheng T.-Y., Crosby W.B., Woolums A.R., Clawson M.L., Chitko-McKown C., Brodersen B., Loy J.D. (2023). Detection of Mannheimia Haemolytica-Specific IgG, IgM and IgA in Sera and Their Relationship to Respiratory Disease in Cattle. Animals.

[14] DelCurto T., Murphy T., Moreaux S. (2017). Demographics and Long-Term Outlook for Western Us Beef, Sheep, and Horse Industries and Their Importance for the Forage Industry. Proceedings of the 47th Western Alfalfa & Forage Symposium.

[15] De Vries A., Overton M., Fetrow J., Leslie K., Eicker S., Rogers G. (2008). Exploring the Impact of Sexed Semen on the Structure of the Dairy Industry. J. Dairy Sci..

[16] Overton M.W., Dhuyvetter K.C. (2020). Symposium Review: An Abundance of Replacement Heifers: What Is the Economic Impact of Raising More than Are Needed?. J. Dairy Sci..

[17] Berry D.P. (2021). Invited Review: Beef-on-Dairy—The Generation of Crossbred Beef × Dairy Cattle. J. Dairy Sci..

[18] McCabe E.D., King M.E., Fike K.E., Odde K.G. (2022). Effects of Holstein and Beef-Dairy Cross Breed Description on the Sale Price of Feeder and Weaned Calf Lots Sold through Video Auctions. Appl. Anim. Sci..

[19] Timsit E., Tison N., Booker C.W., Buczinski S. (2019). Association of Lung Lesions Measured by Thoracic Ultrasonography at First Diagnosis of Bronchopneumonia with Relapse Rate and Growth Performance in Feedlot Cattle. Vet. Intern. Med..

[20] Crawford D.M., Richeson J.T., Perkins T.L., Samuelson K.L. (2022). Feeding a High-Energy Finishing Diet upon Arrival to High-Risk Feedlot Calves: Effects on Health, Performance, Ruminal pH, Rumination, Serum Metabolites, and Carcass Traits. J. Anim. Sci..

[21] Ballou M.A., Davis E.M., Kasl B.A. (2019). Nutraceuticals. Vet. Clin. N. Am. Food Anim. Pract..

[22] Walker W.L., Epperson W.B., Wittum T.E., Lord L.K., Rajala-Schultz P.J., Lakritz J. (2012). Characteristics of Dairy Calf Ranches: Morbidity, Mortality, Antibiotic Use Practices, and Biosecurity and Biocontainment Practices. J. Dairy Sci..

[23] Word A.B., Ellis G.B., Holland B.P., Streeter M.N., Hutcheson J.P. (2021). Effects of Antimicrobial Metaphylaxis Using No Antimicrobial, Tilmicosin, or Tildipirosin and 2 Different Days on Feed on the Health and Growth Performance of Lightweight Beef Steer Calves Originating from Mexico. Appl. Anim. Sci..

[24] Nickell J.S., White B.J. (2010). Metaphylactic Antimicrobial Therapy for Bovine Respiratory Disease in Stocker and Feedlot Cattle. Vet. Clin. N. Am. Food Anim. Pract..

[25] O’Connor A.M., Hu D., Totton S.C., Scott N., Winder C.B., Wang B., Wang C., Glanville J., Wood H., White B. (2019). A Systematic Review and Network Meta-Analysis of Injectable Antibiotic Options for the Control of Bovine Respiratory Disease in the First 45 Days Post Arrival at the Feedlot. Anim. Health Res. Rev..

[26] Stanton A.L., Kelton D.F., LeBlanc S.J., Millman S.T., Wormuth J., Dingwell R.T., Leslie K.E. (2010). The Effect of Treatment with Long-Acting Antibiotic at Postweaning Movement on Respiratory Disease and on Growth in Commercial Dairy Calves. J. Dairy Sci..

[27] Binversie E.S., Ruegg P.L., Combs D.K., Ollivett T.L. (2020). Randomized Clinical Trial to Assess the Effect of Antibiotic Therapy on Health and Growth of Preweaned Dairy Calves Diagnosed with Respiratory Disease Using Respiratory Scoring and Lung Ultrasound. J. Dairy Sci..

[28] Holschbach C.L., Raabis S.M., Ollivett T.L. (2019). Effect of Antibiotic Treatment in Preweaned Holstein Calves after Experimental Bacterial Challenge with Pasteurella Multocida. J. Dairy Sci..

[29] Ollivett T.L., Buczinski S. (2016). On-Farm Use of Ultrasonography for Bovine Respiratory Disease. Vet. Clin. N. Am. Food Anim. Pract..

[30] Goecke N.B., Nielsen B.H., Petersen M.B., Larsen L.E. (2021). Design of a High-Throughput Real-Time PCR System for Detection of Bovine Respiratory and Enteric Pathogens. Front. Vet. Sci..

[31] Apley M.D. (2015). Treatment of Calves with Bovine Respiratory Disease. Vet. Clin. N. Am. Food Anim. Pract..

[32] Collier R.J., Laun W.H., Rungruang S., Zimbleman R.B. (2012). Quantifying Heat Stress and Its Impact on Metabolism and Performance.

[33] Stull C., Reynolds J. (2008). Calf Welfare. Vet. Clin. N. Am. Food Anim. Pract..

[34] Wang J., Li J., Wang F., Xiao J., Wang Y., Yang H., Li S., Cao Z. (2020). Heat Stress on Calves and Heifers: A Review. J. Anim. Sci Biotechnol..

[35] Pereira R.V., Altier C., Siler J.D., Mann S., Jordan D., Warnick L.D. (2020). Longitudinal Effects of Enrofloxacin or Tulathromycin Use in Preweaned Calves at High Risk of Bovine Respiratory Disease on the Shedding of Antimicrobial-Resistant Fecal Escherichia Coli. J. Dairy Sci..

[36] Larson R.L., Step D.L. (2012). Evidence-Based Effectiveness of Vaccination Against Mannheimia Haemolytica, Pasteurella Multocida, and Histophilus Somni in Feedlot Cattle for Mitigating the Incidence and Effect of Bovine Respiratory Disease Complex. Vet. Clin. N. Am. Food Anim. Pract..

[37] Comerford J. Causes of Vaccine Failure in Beef Cattle. https://extension.psu.edu/causes-of-vaccine-failure-in-beef-cattle.

[38] Richeson J.T. Vaccinating High-Risk Calves against BRD. Proceedings of the Forty-Eighth Annual Conference. American Association of Bovine Practitioners.

[39] Arthington J.D., Cooke R.F., Maddock T.D., Araujo D.B., Moriel P., DiLorenzo N., Lamb G.C. (2013). Effects of Vaccination on the Acute-Phase Protein Response and Measures of Performance in Growing Beef Calves1. J. Anim. Sci..

[40] Nowakowski M.A., Inskeep P.B., Risk J.E., Skogerboe T.L., Benchaoui H.A., Meinert T.R., Sherington J., Sunderland S.J. (2004). Pharmacokinetics and Lung Tissue Concentrations of Tulathromycin, a New Triamilide Antibiotic, in Cattle. Vet. Ther..

[41] Crosby S., Credille B., Giguère S., Berghaus R. (2018). Comparative Efficacy of Enrofloxacin to That of Tulathromycin for the Control of Bovine Respiratory Disease and Prevalence of Antimicrobial Resistance in Mannheimia Haemolytica in Calves at High Risk of Developing Bovine Respiratory Disease1. J. Anim. Sci..

[42] Munoz V.I., Samuelson K.L., Tomczak D.J., Seiver H.A., Smock T.M., Richeson J.T. (2020). Comparative Efficacy of Metaphylaxis with Tulathromycin and Pentavalent Modified-Live Virus Vaccination in High-Risk, Newly Received Feedlot Cattle. Appl. Anim. Sci..

[43] Taylor J.D., Fulton R.W., Lehenbauer T.W., Step D.L., Confer A.W. (2010). The Epidemiology of Bovine Respiratory Disease: What Is the Evidence for Predisposing Factors?. Can. Vet. J..

[44] Cusack P., McMeniman N., Lean I. (2003). The Medicine and Epidemiology of Bovine Respiratory Disease in Feedlots. Aust Vet. J.

[45] Padalino B., Cirone F., Zappaterra M., Tullio D., Ficco G., Giustino A., Ndiana L.A., Pratelli A. (2021). Factors Affecting the Development of Bovine Respiratory Disease: A Cross-Sectional Study in Beef Steers Shipped From France to Italy. Front. Vet. Sci..

[46] Cusack P., McMeniman N., Lean I. (2007). Feedlot Entry Characteristics and Climate: Their Relationship with Cattle Growth Rate, Bovine Respiratory Disease and Mortality. Aust. Vet. J..

[47] Diesel D.A., Lebel J.L., Tucker A. (1991). Pulmonary Particle Deposition and Airway Mucociliary Clearance in Cold-Exposed Calves. Am. J. Vet. Res..

[48] Backgrounding Feeder Cattle Nutrition | Cattle. https://www.saskatchewan.ca/business/agriculture-natural-resources-and-industry/agribusiness-farmers-and-ranchers/livestock/cattle-poultry-and-other-livestock/cattle/backgrounding-and-feeder-cattle-nutrition.

[49] Wang S., Li Q., Peng J., Niu H. (2023). Effects of Long-Term Cold Stress on Growth Performance, Behavior, Physiological Parameters, and Energy Metabolism in Growing Beef Cattle. Animals.

[50] Committee on Nutrient Requirements of Dairy Cattle, Board on Agriculture and Natural Resources, Division on Earth and Life Studies, National Academies of Sciences, Engineering, and Medicine (2021). Nutrient Requirements of Dairy Cattle: Eighth Revised Edition.

[51] Roland L., Drillich M., Klein-Jöbstl D., Iwersen M. (2016). Invited Review: Influence of Climatic Conditions on the Development, Performance, and Health of Calves. J. Dairy Sci..

[52] Patterson D.J., Bellows R.A., Burfening P.J., Carr J.B. (1987). Occurrence of Neonatal and Postnatal Mortality in Range Beef Cattle. I. Calf Loss Incidence from Birth to Weaning, Backward and Breech Presentations and Effects of Calf Loss on Subsequent Pregnancy Rate of Dams. Theriogenology.

[53] Cuevas-Gómez I., McGee M., Sánchez J.M., O’Riordan E., Byrne N., McDaneld T., Earley B. (2021). Association between Clinical Respiratory Signs, Lung Lesions Detected by Thoracic Ultrasonography and Growth Performance in Pre-weaned Dairy Calves. Ir. Vet. J..

[54] Sáadatnia A., Mohammadi G.R., Azizzadeh M., Mirshahi A., Mohieddini A.A., Buczinski S. (2023). Effect of Ultrasonographic Lung Consolidation on Health and Growth in Dairy Calves: A Longitudinal Study. J. Dairy Sci..

[55] Rhodes V., Ryan E.G., Hayes C.J., McAloon C., O’Grady L., Hoey S., Mee J.F., Pardon B., Earley B., McAloon C.G. (2021). Diagnosis of Respiratory Disease in Preweaned Dairy Calves Using Sequential Thoracic Ultrasonography and Clinical Respiratory Scoring: Temporal Transitions and Association with Growth Rates. J. Dairy Sci..

[56] Snyder E., Credille B., Berghaus R., Giguère S. (2017). Prevalence of Multi Drug Antimicrobial Resistance in Mannheimia Haemolytica Isolated from High-Risk Stocker Cattle at Arrival and Two Weeks after Processing1. J. Anim. Sci..

[57] Woolums A.R., Karisch B.B., Frye J.G., Epperson W., Smith D.R., Blanton J., Austin F., Kaplan R., Hiott L., Woodley T. (2018). Multidrug Resistant Mannheimia Haemolytica Isolated from High-Risk Beef Stocker Cattle after Antimicrobial Metaphylaxis and Treatment for Bovine Respiratory Disease. Vet. Microbiol..

