# Supplementary material for: Bovine Respiratory Disease (BRD) in Post-Weaning Calves with Different Prevention Strategies and the Impact on Performance and Health Status
Source: Animals (Basel). 2024 Sep 28;14(19):2807. doi: 10.3390/ani14192807 (PMC11476203; doi:10.3390/ani14192807)
Supplement: Supplementary file 1 [file animals-14-02807-s001.zip › animals-3169465-supplementary.pdf]

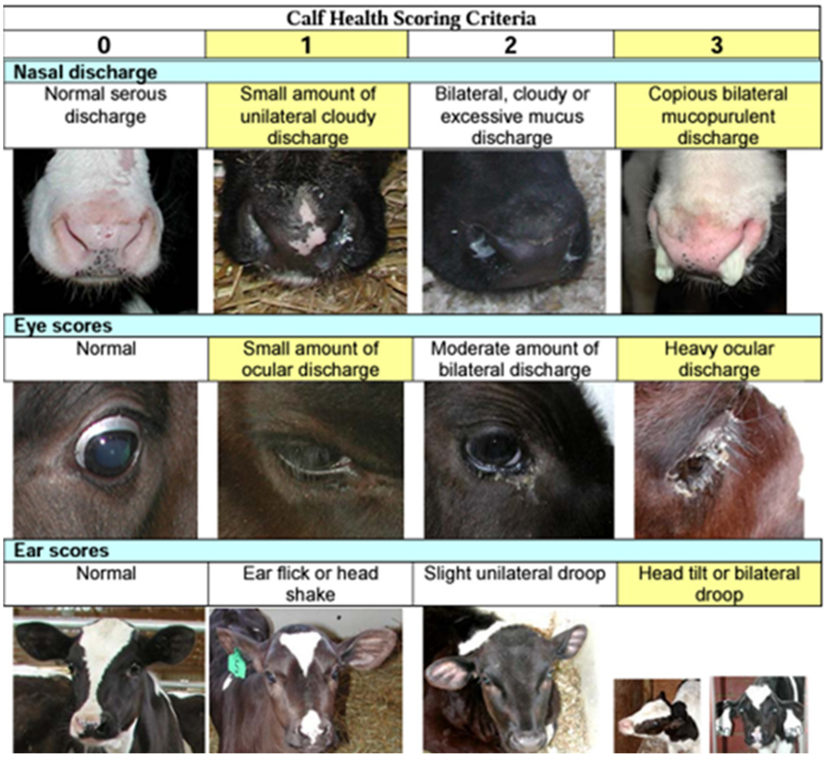

Figure S1. The scoring system used in the study was adapted from the Calf Health Scoring Chart from the University of Wisconsin, a total score of 9 was possible (nasal discharge, eyes, and ears).
